# Urinary Incontinence Due to Complete Ureteral Duplication With an Ectopic Vulvar Ureteral Orifice: A Case Report

**DOI:** 10.7759/cureus.42114

**Published:** 2023-07-19

**Authors:** Catarina Pioli Lamêgo de Faria, Lara Gomes Botelho, Isadora Santos Vidal, Alexander Hatsumura Casini, Antônio Chambô Filho

**Affiliations:** 1 Department of Gynecology, Santa Casa de Misericórdia Hospital, Vitória, BRA; 2 Department of General Surgery, Santa Casa de Misericórdia Hospital, Vitória, BRA

**Keywords:** congenital malformation, abnormal ureteral insertion, urinary incontinence, ectopic ureter, ureteral duplication

## Abstract

Ureteral duplication is one of the most common congenital malformations of the urinary tract and may be complete or incomplete. One of the complications of complete ureteral duplication is an ectopic ureter orifice, which, depending on the opening site, may cause urinary incontinence in females, a condition with potentially serious repercussions on the woman’s quality of life. Thus, the present study aims to report the case of a 24-year-old female patient with a complaint of urinary incontinence since childhood. After a physical examination and imaging tests, she was diagnosed with complete ureteral duplication on the left side, associated with sequelar parenchymal atrophy of the upper pole of the left kidney, and ectopic vulvar ureter. The patient underwent a videolaparoscopic left upper polar nephrectomy, and her symptoms improved after surgery. This report intends to add to already available data in the literature, highlighting the relevance of anamnesis and physical examination in reaching a diagnosis and implementing appropriate treatment, thus improving the quality of life of individuals with this condition. In addition, these data should be useful both for the medical community and for future studies on this malformation.

## Introduction

Urinary incontinence is characterized by the involuntary loss of any volume of urine, and it has a variable prevalence that increases with age. It is a condition that has various implications for a woman's quality of life [1,2,3]. Urinary incontinence can be classified as urethral and extra-urethral, with the latter caused by urinary fistulas or congenital malformations such as the ectopic ureter, which is a very rare anomaly with an incidence of 0.05% to 0.025% [4,5]. Ureteral disorders can be classified as congenital or acquired [6]. Ureteral duplication is one of the most common congenital malformations of the urinary tract, occurring more frequently in females and can be complete or incomplete [6,7].

In incomplete ureteral duplication, there are two separate collecting systems and two ureters that can join together at any level between the kidney and the bladder, forming a single ureter that opens normally into the bladder base [7]. In complete ureteral duplication, the presence of two ureteric buds leads to the formation of two ureters and two completely separate renal pelvises [6].

The most frequent associated anomaly in complete ureteral duplication is ureterocele. Other complications include vesicoureteral reflux, ureteropelvic junction obstruction, ureters with ectopic openings, and megaureter [7,8,9,10]. Many patients with ureteral duplication are asymptomatic; however, a common presentation is persistent or recurrent urinary tract infections or even urinary incontinence with preserved voiding (exclusively in females) [6,7].

The objective of the present report was to describe the case of a female patient with urinary incontinence due to complete ureteral duplication with an ectopic ureter opening into the vulva. This report should contribute toward facilitating the identification of this pathology, increasing the available information on the subject, and improving treatment management.

## Case presentation

A 24-year-old woman with two previous pregnancies resulting in two vaginal deliveries presented at the gynecology clinic of a tertiary hospital complaining of constant loss of urine since childhood that was unrelated to physical activity or intra-abdominal pressure. She reported normal voiding associated with episodes of recurrent urinary tract infections and back pain on the left side. She had to resort to using sanitary protection in her underwear due to the condition, limiting her social life and involving psychological and socioeconomic costs. General practitioners and medical specialists (pediatricians and urologists) had previously failed to clarify the etiology of the condition.

**Figure 1:**
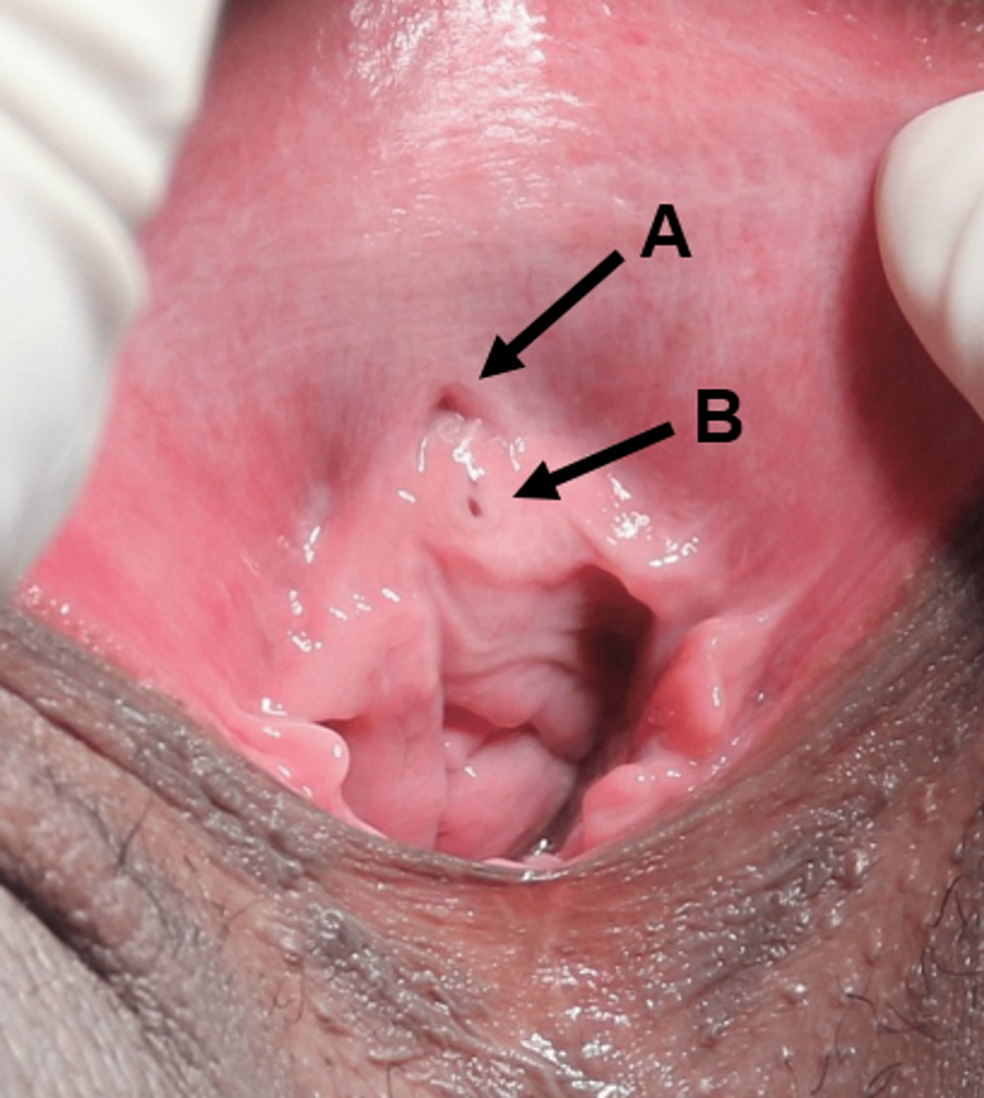
Physical examination showing topical (A) urethral meatus and, below, the (B) ectopic ureteral orifice.

Physical examination showed a topical urethral meatus and the presence of an orifice around 1 cm below the urethra, with visible emission of urine. The hypothetical diagnosis was the ectopic insertion of the ureter (Figure 1).

**Figure 2:**
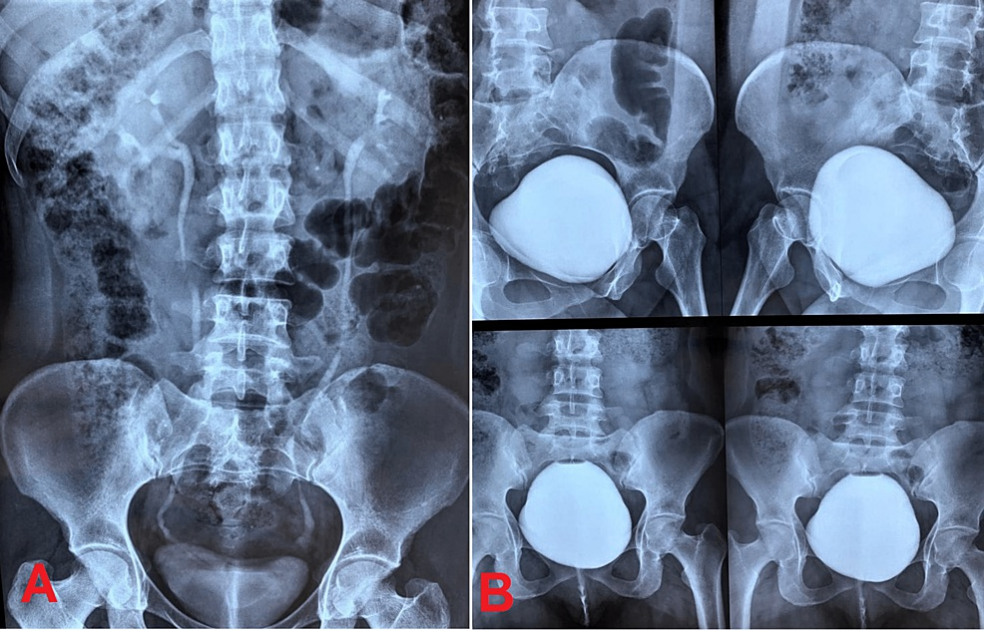
(A) Excretory urography and (B) a voiding cystourethrography, neither revealing any abnormalities.

**Figure 3:**
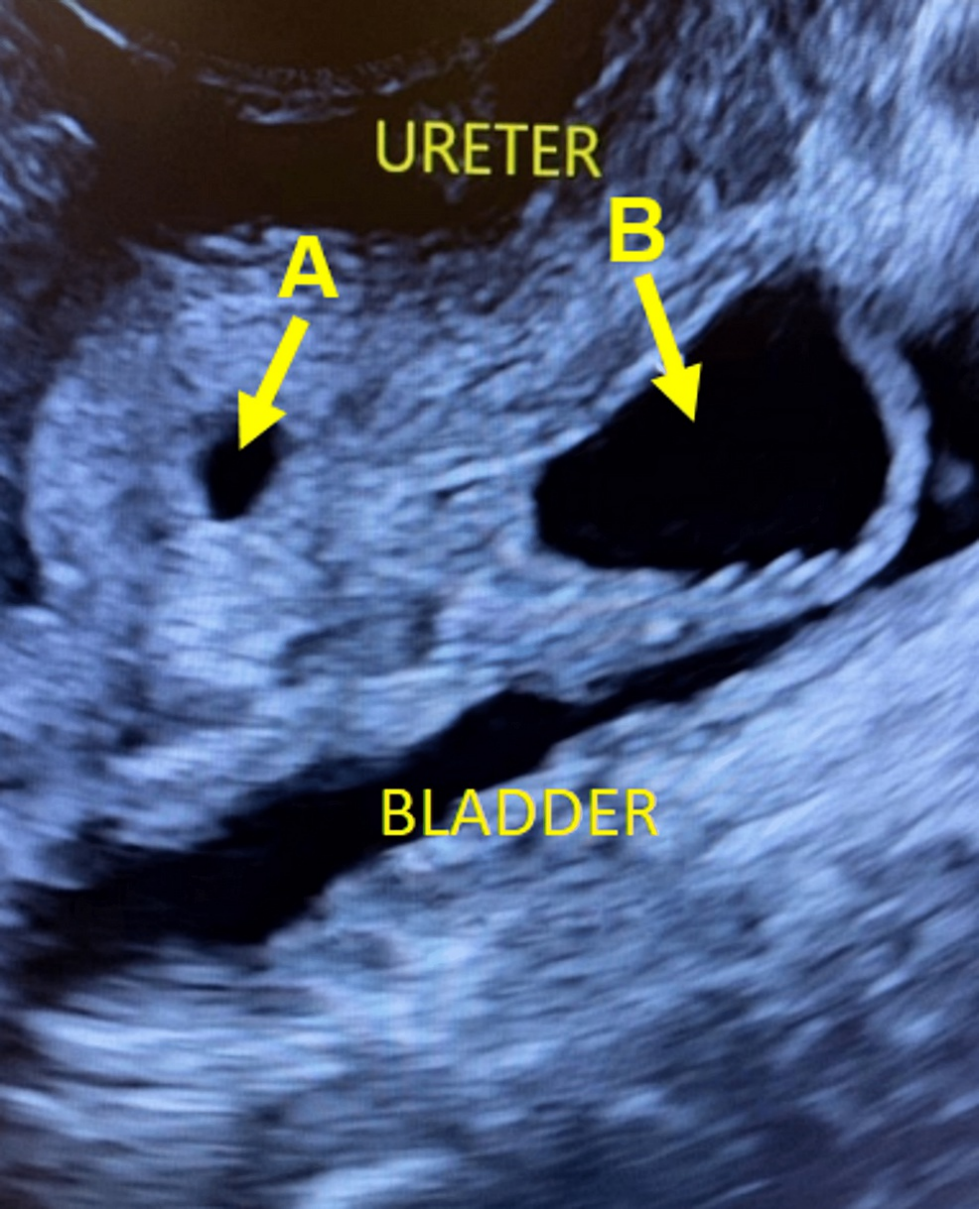
Kidney and urinary tract ultrasound image showing ureteral duplication with (A) a ureter of normal caliber and (B) ectopic ureter of enlarged caliber/ectasia. The bladder was not full.

**Figure 4:**
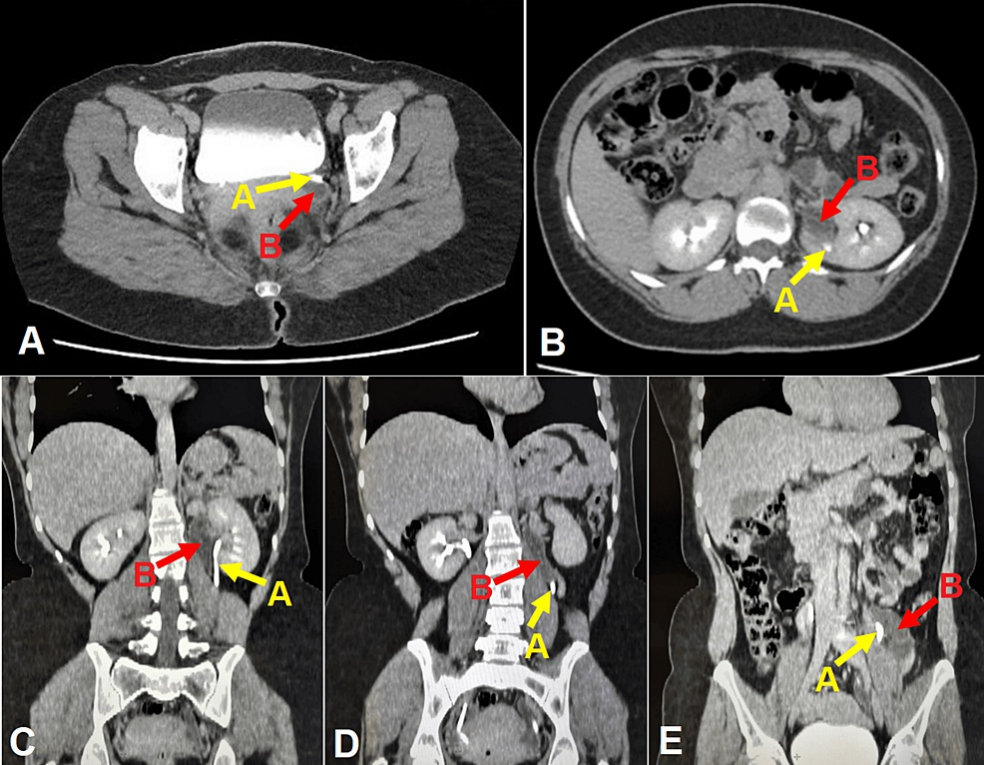
Computed tomography showing duplicated pyelocaliceal system and complete duplication of the ureter on the left side, with dilated ectopic ureter associated with consequent parenchymal atrophy of the upper pole of the left kidney. (A) and (B) A (yellow arrow) indicates a ureter of normal caliber and B (red arrow) an enlarged ectopic ureter; (C)-(E) red arrow B indicates the proximal, medial, and distal ends of the ureteral duplication, respectively, and yellow arrow A indicates the normal ureter.

Imaging tests carried out for clarification included an excretory urography and a voiding cystourethrography, neither revealing any abnormalities (Figure 2). Kidney and urinary tract ultrasound images showed ureteral duplication and abnormal insertion of the ureter with ureteral ectasia (Figure 3). Computed tomography of the abdomen showed duplicated pyelocaliceal system and complete duplication of the ureter on the left side (anatomical variation) with ectasia of the collecting system draining the upper pole of the left kidney, associated with consequent renal parenchymal atrophy and lack of opacity in the respective collecting system. There was no evidence of dilatation of the other pyelocaliceal systems or of kidney stones. The ureters were patent, of normal size, and following their normal path (Figure 4). Despite the absence of opacity in the collecting system with respect to the upper pole of the left kidney, ureteral dilatation was found up to the region corresponding to the vulva (the site of the abnormal insertion of the respective ureter).

**Figure 5:**
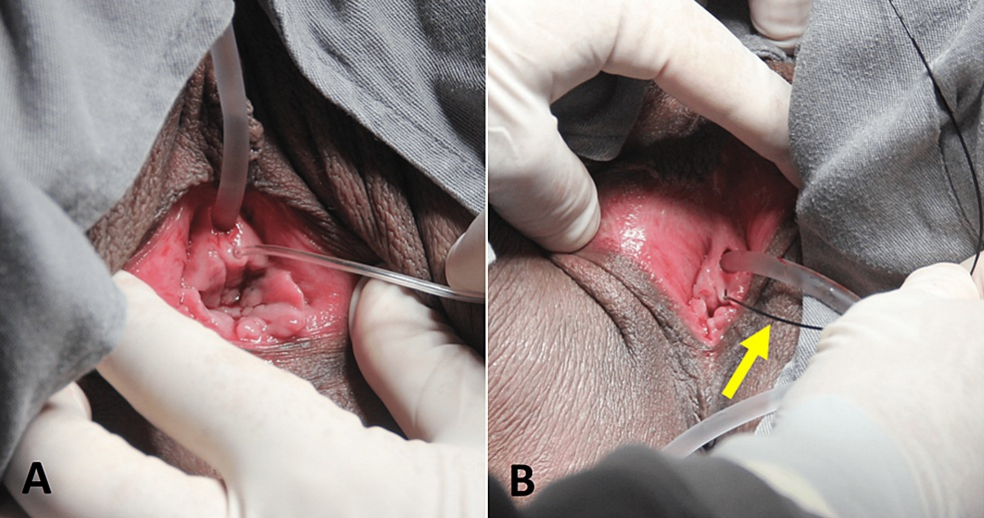
(A) and (B) Catheterization using a guide wire into the ectopic ureteral meatus, as indicated by the arrow in (B).

Based on those findings, the patient was seen by the urology team, and a cystoscopy was performed. A normal ureteral meatus was found on the right side and only one ureteral meatus was detected on the left side. Ureteral catheterization was performed on the left side together with pyelography, revealing a normal collecting system and no dilatation. Catheterization was then performed using a guide wire into the ectopic ureteral meatus, below the urethral meatus, advancing the guide wire up to the upper pole of the kidney that was responsible for the abnormal urine leakage and ectopic insertion of the ureter (Figure 5). These findings confirmed the diagnosis of complete ureteral duplication on the left side, with the upper pole of the left kidney being almost completely excluded and the drainage from the abnormal ureter being diverted to the vulva.

**Figure 6:**
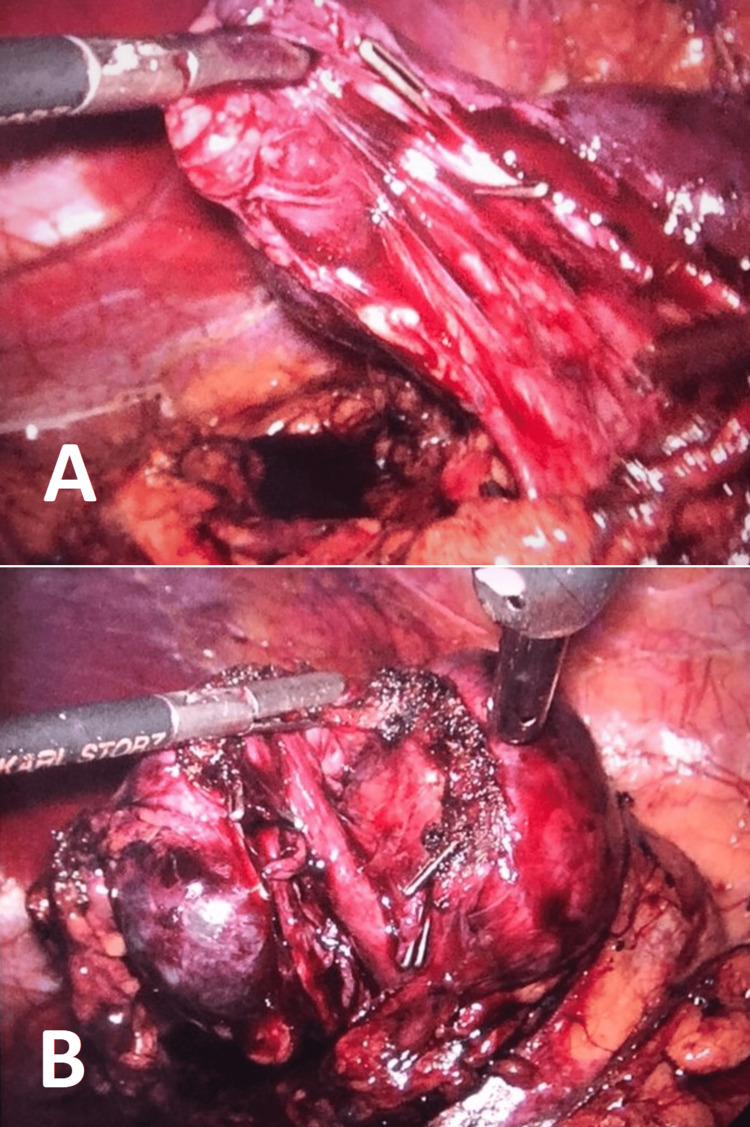
(A) and (B) Video-laparoscopy with partial nephrectomy of the upper pole of the left kidney.

The patient then underwent elective surgery, with upper pole partial nephrectomy being performed by video-laparoscopy, which proceeded with no complications (Figure 6). Histopathology of the surgical specimen, stained with hematoxylin and eosin, showed nonspecific chronic pyelonephritis, tubular atrophy, glomerulosclerosis, and hydronephrosis.

The patient progressed without any complications following surgery and was able to return to work two weeks later. A year after the procedure, she reported having had no episodes of urinary incontinence, only one episode of urinary tract infection, and a significant improvement in her quality of life.

## Discussion

In children, daytime urinary continence is usually achieved before four years of age, while nocturnal continence starts before five years of age [11]. After that early phase of life, any involuntary loss of urine characterizes urinary incontinence and can be continuous, occurring during the day and at night, or intermittent. Identifying whether incontinence is continuous or intermittent is important in defining etiology, as when urine loss is continuous, anatomical causes need to be confirmed or ruled out such as ectopic ureter, which is a very rare anomaly with an incidence of 0.05% to 0.025%, occurring mainly in women. It is characterized by the opening of the ureter in an area outside the posterolateral end of the bladder trigone, and most cases are associated with complete ureteral duplication [5,12,13]. This diagnosis should preferably be made in childhood since as age progresses, other causes of urinary incontinence become more predominant, and as a consequence, the diagnosis of ectopic ureter may be forgotten or delayed [14]. In our case, we present a young patient who had continuous urinary incontinence since childhood, persisting into adulthood, and the diagnosis was delayed due to inadequate evaluation. The hypothesis of anomalous ureteral insertion was only considered after a thorough anamnesis and physical examination had been conducted.

The ureter is a complex functional conduit responsible for channeling urine from the kidneys to the bladder. Kidney abnormalities can occur if any pathologic process interferes with this activity. While disorders of the ureter can be classified as congenital or acquired [6], ureteral duplication is one of the most common congenital malformations of the urinary tract, occurring in about 0.8% of the population, and can be complete or incomplete. This condition is more common in females than in males, and it is more often unilateral than bilateral [6,7,15].

In incomplete ureteral duplication, there are two collecting systems and two ureters. The two ureters join together at any level between the kidney and the bladder, in most cases in the lower third of that trajectory, to form a single ureter that drains normally into the bladder base [7]. In complete ureteral duplication, on the other hand, there are two ureters and two completely separate renal pelvises. Since the ureter for the upper segment originates from a position above the mesonephric duct, it remains attached to this duct for longer and consequently migrates forward, establishing a medial position lower than the ureter that drains the lower segment (Weigert-Meyer law). Therefore, the ureter that drains the upper segment may migrate farther in a caudal direction, becoming ectopic and obstructed, while the ureter that drains the lower segment may terminate laterally with a short intravesical tunnel that more often results in vesicoureteral reflux [6]. Complete ureteral duplication can also be associated with increased pressure in the upper renal parenchyma, which can lead to atrophy and loss of function in this unit [10]. In our patient, after adequate investigation, it was identified that she had complete ureteral duplication on the left side, with the duplicated ureter corresponding to the upper pole of the left kidney and ectopic opening in the vulva, about 1 cm below the urethra, following the Weigert-Meyer law. She also had sequelar parenchymal atrophy of the upper pole of the left kidney.

In cases of ectopic ureter associated with ureteral duplication, the grade of ectopia can range from a position just slightly below the topic meatus to extravesical implantation, with the clinical characteristics of the condition presented in these cases depending on the sex of the patient [8]. In females, the ureter for the upper pole can be ectopic, with an opening that is distal from the external sphincter or even outside the urinary tract. In these patients, symptoms consist of urinary incontinence characterized by continuous dripping and, simultaneously, delayed voiding. In males, there is no incontinence because the ectopic ureteral meatus is always situated above the external urethral sphincter [6,8]. Our patient's main complaint was continuous urinary incontinence since childhood, resulting from continuous urine leakage through the vulvar ectopic ureter, despite reporting normal voiding.

The imaging tests used for diagnosis include ultrasonography, which in recent years has led to the diagnosis of many asymptomatic infants; voiding urethrocystography, which is important for determining the presence of vesicoureteral reflux and confirming the presence of a ureterocele; and renal scintigraphy, particularly Tc-99m dimercaptosuccinic acid (DMSA) scintigraphy, which is useful for measuring renal function in each segment of the kidney [6]. Furthermore, with the advent of computed tomography and magnetic resonance imaging, these tests began to be widely used to clarify diagnosis and aid treatment of disorders of the kidneys and urinary tract. Over the past 10 years, computed tomography has overtaken excretory urography for the evaluation of the genitourinary tract and is currently one of the most commonly used tests [7]. In our case, the complementary exams responsible for the diagnosis of complete ureteral duplication with vulvar ectopic ureter were renal and urinary tract ultrasound, followed by abdominal computed tomography and cystoscopy. Excretory urography did not show any abnormalities, as the upper pole of the left kidney (associated with ureteral duplication) was functionally excluded and did not excrete contrast. Computed tomography also did not show contrast excretion by the parenchyma of the upper pole of the left kidney, confirming atrophy and loss of function in this unit.

Due to the anatomical variations, a range of surgical options is available, depending on the functionality of the renal portion drained by the ectopic ureter [5]. If the upper segment is obstructed, surgery is almost always necessary. If renal function is much reduced in one segment, partial nephrectomy is the most appropriate procedure. Treatments involving pyeloureterostomy, uretero-ureterostomy, and ureteral reimplantation are recommended to preserve the renal parenchyma when renal function is still adequate. These treatments can be performed by laparoscopy or open surgery [6].

Wang performed a heminephroureterectomy on the nonfunctional upper pole of a kidney and obtained good results [13]. In another study, Demirtas et al. performed a ureteroureterostomy, as the patient's renal functional capacity was sufficient for preserving the upper pole, with satisfactory results. They preferred this surgery over ureteroneocystostomy as it does not require an incision in the bladder [5]. Wong and Braga performed a distal ipsilateral ureteroureterostomy [16]. In our patient, due to the atrophy and significant loss of function in the upper pole of the left kidney associated with ureteral duplication, we opted for upper pole partial nephrectomy being performed by video-laparoscopy, which occurred without complications and yielded good results, as the patient showed improvement in symptoms after surgery.

## Conclusions

Complete ureteral duplication is a congenital malformation associated with ectopic implantation of the ureter. This condition involves a range of consequences that include urinary incontinence, a disorder with many repercussions on the patient’s health and well-being. This paper describes the case of a young woman who had had urinary incontinence since childhood and was found to have abnormal implantation of the ureter. She had already sought help from various professionals; however, the correct diagnosis had yet to be reached and adequate treatment for this pathology implemented, with direct consequences to her quality of life, and physical and emotional health. Because urinary incontinence can be urethral or extra-urethral, a detailed anamnesis and appropriate physical examination are vital in determining a diagnostic hypothesis and an appropriate investigation with supplementary tests required to reach a diagnosis followed by selection of a suitable surgical treatment.
